# Vector control using LLIN/ITN: reduction of malaria morbidity in Bangladesh

**DOI:** 10.1186/1475-2875-13-S1-P47

**Published:** 2014-09-22

**Authors:** Mohammad Moktadir Kabir, Shamsun Naher, Akramul Islam, Abdul Karim, Mohammad Harun-Or Rasid, Shahidul Islam Laskar

**Affiliations:** 1 BRAC Health, Nutrition and Population Programme, Dhaka, Bangladesh

## 

Malaria is one of the major public health concerns due to re-emergence of the disease in Bangladesh in the 1990s. Currently it is endemic in 13 out of 64 administrative districts bordering India and Myanmar. Three hill tract districts (Khagrachhari, Rangmati, Bandarban) in the south-eastern part of the country report the highest incidences. The overall prevalence rate is 3.1% in the 13 endemic districts and 11% in 3 hill districts.

BRAC initiated a pilot malaria control project in one of these hill districts in 1998. By 2004, the program expanded to all three hill tract districts covering 1.4 million population with the support from National Malaria Control Programme and with technical support from WHO. Later in 2007, BRAC led consortium of 20 partner NGOs started comprehensive implementation of the programme in all endemic districts with a population of 11 million in collaboration with the government and with financial support from the Global Fund to fight AIDS, TB and Malaria (GFATM). The aim was to control the vector, to prevent disease transmission, and to provide early diagnosis and prompt effective treatment services to reduce morbidity and mortality.

Currently Long Lasting Insecticidal Nets (LLIN) are being distributed and ordinary bed nets are being treated with insecticide (Insecticide Treated Net- ITN) to the endemic districts. BRAC has selected female Community Health Workers from their own community and involved them to reach the poor and people living in areas with limited access to treatment services supplementing the government’s health care system. They provide home-based care of malaria diagnosis and treatment. They also provide malaria related information especially on malaria transmission, bed net usage, malaria symptoms and available treatment services at community level. Different sensitization meetings are conducted with opinion and religious leaders, local government and civil society representatives to increase awareness on malaria prevention and management. A recent survey showed that net utilization rate among children under five is 90% and among pregnant women is 85% in malaria endemic region. The national disease specific report shows a substantial decline in malaria cases from the baseline year 2008 (84,690 cases) to 2013 (26,891 cases), a 68% reduction of cases.

Increased use rate of LLIN/ITN along with early seeking of home based malaria management is the key to success in malaria control.

